# Cervicovaginal microbiome and natural history of HPV in a longitudinal study

**DOI:** 10.1371/journal.ppat.1008376

**Published:** 2020-03-26

**Authors:** Mykhaylo Usyk, Christine P. Zolnik, Philip E. Castle, Carolina Porras, Rolando Herrero, Ana Gradissimo, Paula Gonzalez, Mahboobeh Safaeian, Mark Schiffman, Robert D. Burk, Costa Rica HPV Vaccine Trial (CVT) Group

**Affiliations:** 1 Department of Pediatrics (Genetic Medicine), Albert Einstein College of Medicine, Bronx, New York, United States of America; 2 Department of Epidemiology and Population Health, NYU School of Medicine, New York, New York, United States of America; 3 Department of Biology, Long Island University, Brooklyn, New York, United States of America; 4 Department of Epidemiology and Population Health, Albert Einstein College of Medicine, Bronx, New York, United States of America; 5 Agencia Costarricense de Investigaciones Biomédicas (ACIB), formerly Proyecto Epidemiológico Guanacaste, Fundación INCIENSA, San José, Costa Rica; 6 Prevention and Implementation Group, International Agency for Research on Cancer, Lyon, France; 7 Roche Molecular Diagnostics, Pleasanton, California, United States of America; 8 Division of Cancer Epidemiology and Genetics (DCEG), National Cancer Institute, NIH, Bethesda, Maryland, United States of America; 9 Departments of Microbiology and Immunology, and Obstetrics and Gynecology and Women’s Health, Albert Einstein College of Medicine, Bronx, New York, United States of America; Emory University, UNITED STATES

## Abstract

**Background:**

Human papillomavirus (HPV) infection is one of the most common sexually transmitted infections. However, only a small percentage of high-risk (HR) HPV infections progress to cervical precancer and cancer. In this study, we investigated the role of the cervicovaginal microbiome (CVM) in the natural history of HR-HPV.

**Methods:**

This study was nested within the placebo arm of the Costa Rica HPV Vaccine Trial that included women aged 18–25 years of age. Cervical samples from two visits of women with an incident HR-HPV infection (n = 273 women) were used to evaluate the prospective role of the CVM on the natural history of HR-HPV. We focus specifically on infection clearance, persistence, and progression to cervical intraepithelial neoplasia grade 2 and 3 (CIN2+). The CVM was characterized by amplification and sequencing the bacterial 16S V4 rRNA gene region and the fungal ITS1 region using an Illumina MiSeq platform. OTU clustering was performed using QIIME2. Functional groups were imputed using PICRUSt and statistical analyses were performed using R.

**Results:**

At Visit 1 (V1) abundance of *Lactobacillus iners* was associated with clearance of incident HR-HPV infections (Linear Discriminant Analysis (LDA)>4.0), whereas V1 *Gardnerella* was the dominant biomarker for HR-HPV progression (LDA>4.0). At visit 2 (V2), increased microbial Shannon diversity was significantly associated with progression to CIN2+ (p = 0.027). Multivariate mediation analysis revealed that the positive association of V1 *Gardnerella* with CIN2+ progression was due to the increased cervicovaginal diversity at V2 (p = 0.040). A full multivariate model of key components of the CVM showed significant protective effects via V1 genus *Lactobacillus*, OR = 0.41 (0.22–0.79), V1 fungal diversity, OR = 0.90 (0.82–1.00) and V1 functional Cell Motility pathway, OR = 0.75 (0.62–0.92), whereas V2 bacterial diversity, OR = 1.19 (1.03–1.38) was shown to be predictive of progression to CIN2+.

**Conclusion:**

This study demonstrates that features of the cervicovaginal microbiome are associated with HR-HPV progression in a prospective longitudinal cohort. The analyses indicated that the association of *Gardnerella* and progression to CIN2+ may actually be mediated by subsequent elevation of microbial diversity. Identified features of the microbiome associated with HR-HPV progression may be targets for therapeutic manipulation to prevent CIN2+.

**Trial registration:**

ClinicalTrials.gov NCT00128661.

## Author summary

Despite being the most common sexually transmitted infection and the causal agent of cervical cancer, it is still not clear why only a small proportion of high-risk HPV (HR-HPV) infections progress to cervical cancer. Our study utilizes longitudinal cervicovaginal samples from a prospective cohort, along with advanced epidemiological modeling and mediation analysis, to investigate the association between the cervicovaginal microbiome (CVM) and progression of an incident HR-HPV infection to cervical precancer. The results of our study suggest a novel association between the effect of *Gardnerella* and disruption of CVM homeostasis that can influence the pathway of HR-HPV infection progression to cervical precancer. We further show the interplay between several key components of the cervicovaginal microbiome and demonstrate that within the context of HR-HPV natural history the effect of *Gardnerella* is mediated by increased cervicovaginal bacterial diversity directly preceding the progression of a persistent infection to precancer.

## Introduction

**Fig 1:**
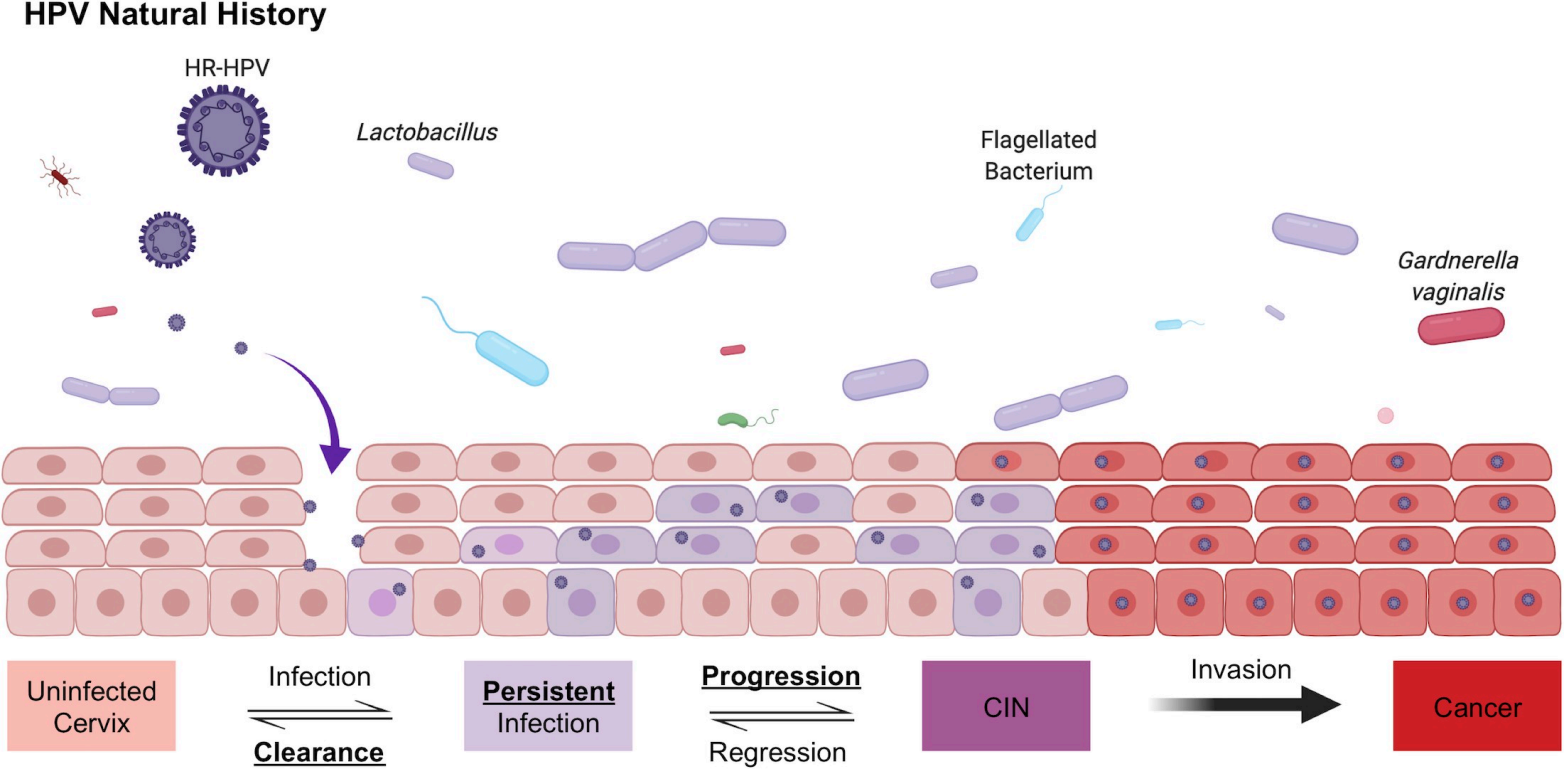
HPV natural history. The natural history of HR-HPV is depicted. Briefly, an incident HR-HPV infection can occur by entering the basal layer through an epithelial abrasion. Most incidence infections are cleared, however some remain persistent for years and decades. Persistence of a HR-HPV infection combined with known risk factors (e.g., smoking) may allow the persistent HR-HPV infection to progress to precancer (cervical intraepithelial neoplasia, CIN). If the lesion does not regress and the HR-HPV is able to successfully integrate into the host-cell genome, clonal expansion may occur and result in an invasive cancer.

Persistent cervical infections by high-risk (HR) human papillomavirus (HPV) cause virtually all cervical cancers and their immediate precursor lesions [1]. Most sexually active women have been infected with HPV at some point in their lives and in the vast majority the infection is cleared within a few months [2]. However, a subset of women develop a persistent HPV infection that places them at high risk for cervical precancer and cancer [2–5]. **Fig 1** illustrates this paradigm canonically referred to as HPV natural history.

Non-viral factors (HPV co-factors) associated with the outcomes of HR-HPV infections have not been fully elucidated. While smoking [6–9], hormonal contraceptive use [10, 11], and parity [12] are associated with developing precancer and cancer; systemic and local immune responses are thought to be important for clearance and control of infection (persistence vs. clearance) [13, 14]. In addition, specific host immune regulatory alleles (e.g., human leukocyte antigen) are associated with risk of cervical cancer [15, 16].

The local, cervical microenvironment, including the microbiome, may also influence the natural history of HPV infection [17]. Other studies have recently implicated the microbiome’s role in the natural history of other viral infections [18] and a variety of cancers [19–21]. The cervicovaginal microbiome (CVM) is of particular interested because it has been well characterized and specific features have been associated with gynecologic disease and reproductive health [22–24]. The CVM has been categorized into community state types (CSTs) generally defined by a dominance of a specific *Lactobacillus* species (*Lactobacillus crispatus*, *Lactobacillus iners*, *Lactobacillus gasseri* or *Lactobacillus jensenii*), or a state of polymicrobialism [25, 26]. Transitions from *Lactobacillus* dominated *CSTs* have been linked to detrimental health outcomes including elevated risks for sexually transmitted infections [27], as well as higher incidences of preterm births [28].

An association between increased CVM diversity and prevalence of HR-HPV infection and/or cervical abnormalities (vs. HPV negative) has been reported in several studies [29–33]. Higher abundance of *L*. *crispatus* has been shown to be associated with lower HPV prevalence [34] and increased detection of normal cytology [35]. Long-term use of vaginal probiotics with *Lactobacillus* spp. has been associated with increase clearance of HPV compared to short-term use [36]. However, evidence is conflicting on the association of CVM diversity and the severity of cervical neoplasia [37–39]. Additionally, most studies looking at the natural history of HPV and the microbiome are cross-sectional and therefore lack the ability to draw potential causal links.

For the current study, we leveraged longitudinal data and specimens from the placebo arm of a large randomized HPV vaccine trial [40] to examine the impact of the CVM on the natural history of incident HR-HPV infections to study: 1) progression to cervical precancer, 2) viral persistence, and 3) viral clearance.

## Results

### Subject characteristics and cervicovaginal microbiome features

A total of 273 women with an incident HR-HPV infection were included in the analyses, of whom 266 had a second sample with an average sampling interval of 1.5±0.9 years. **Table 1** presents the sample demographic information and summarizes the bacterial and fungal sequencing results after taxonomic assignment for each infection outcome at baseline. There were no significant differences between groups in age (p = 0.13), 16S rRNA gene OTU clustered read counts (p = 0.33), or ITS1 OTU clustered read counts (p = 0.53).

**Table 1:** CVT cohort characteristics.

Variable	Clearance	Persistence	Progression	p-val
*Baseline Sample Count*	*70*	*170*	*33*	
Age (years)	23.4 ± 2.8	22.6 ± 2.3	22.70 ± 2.9	0.13
Current/former Smoker	15.7% (11/70)	27.1% (46/170)	26.7% (8/30)	0.16
Total number of sexual partners as of visit	3 ± 3	3 ± 2	3 ± 2	0.63
Contraceptive use*	97.1% (68/70)	97.6% (166/170)	96.7% (29/30)	0.86
Condom use prior to visit	44.1% (15/34)	39.3% (33/84)	58.3% (7/12)	0.46
Contraceptive pill prior to visit	68.8% (33/48)	68.5% (76/111)	52.6% (10/19)	0.40
Injectable contraceptive prior to visit	14.8% (8/54)	21.7% (26/120)	23.8% (5/21)	0.53
Other contraceptive prior to visit**	4.6% (3/65)	0% (0/150)	0% (0/26)	0.029
Chlamydia/Gonorrhea Positive	46.2% (6/13)	30.4% (14/46)	0% (0/3)	0.32
HPV16 Positive	37.1% (26/70)	34.7% (59/170)	45.5% (15/33)	0.49
16SV4 Reads	10,642 ± 4,910	11,482 ± 4,985	11,041 ± 4,809	0.33
ITS1 Reads	1,942 ± 3,433	3,299 ± 7,055	4,660 ± 11,500	0.53

Continuous data is presented using median ± standard deviation, significance assessed using the Kruskal-Wallis test.

Count data is presented using percent with proportions shown in parenthesis, significance is assessed using Fisher's exact test.

Proportion of count data is based on only the samples which had data for a given count variable.

*Counts use of any of the following prior to the visit: condom, birth control pills, diaphragm, injectable, iud, spermicide, sponge and or other** types of contraceptives.

**Fig 2:**
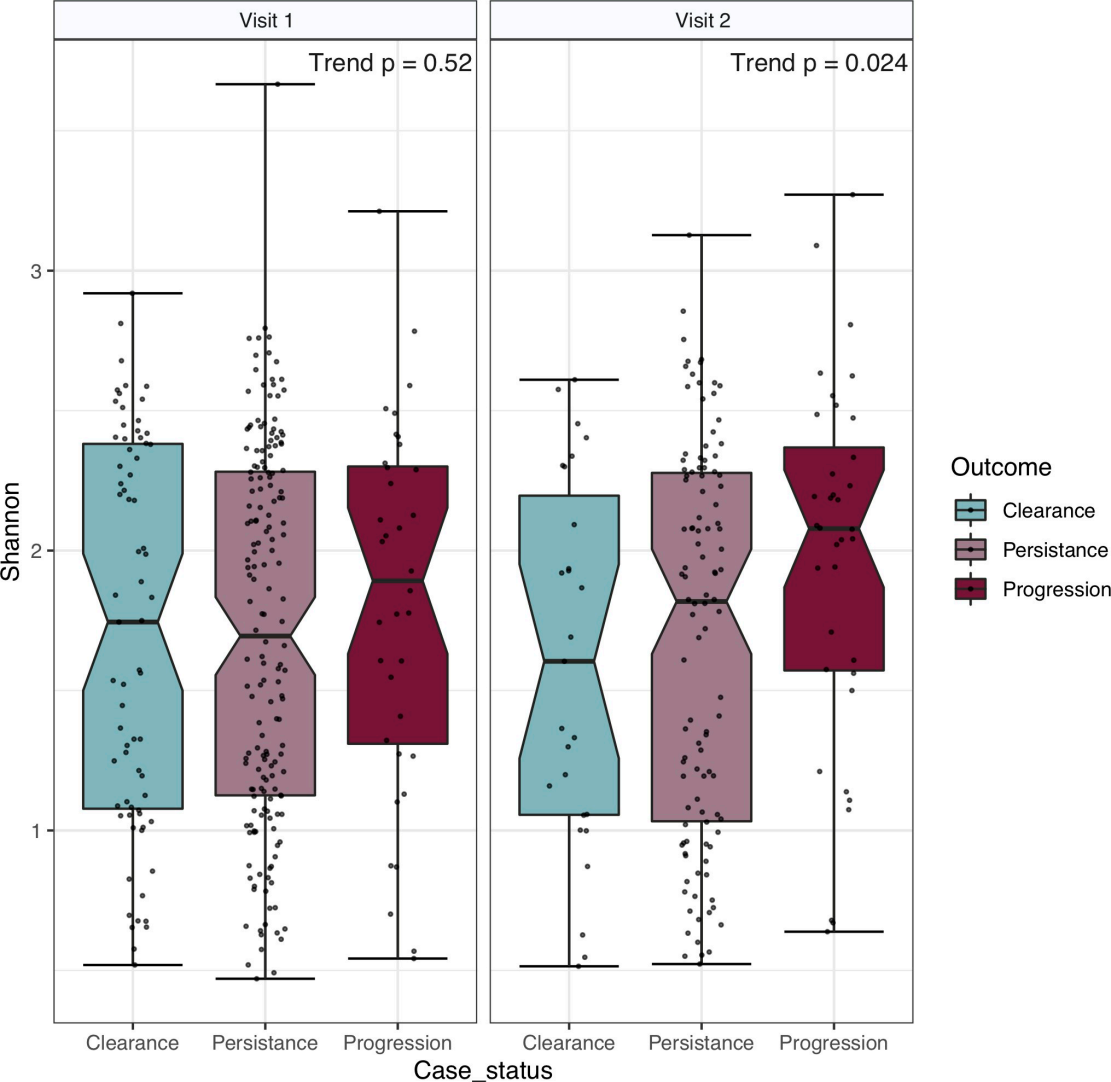
Bacterial Shannon diversity by visit. HR-HPV category specific microbial Shannon diversity is shown for V1 and V2. Horizontal strip labels at the top of the figure indicate visit number. V1 has an elevated diversity in the progression group, but the overall trend did not achieve statistical significance (p = 0.52). At V2 the observed trend of a rising Shannon alpha diversity from clearance to persistence to progression was statistically significant, p = 0.024.

**Fig 2** summarizes the bacterial Shannon diversity measures of the three ordered categorical HR-HPV outcomes (see **Fig 1**) within the CVT cohort at V1 and V2. Alpha diversity analysis did not reveal any significant differences at V1 in terms of bacterial Shannon alpha diversity (trend p = 0.52). At V2 there was a significant trend of rising diversity based on the Shannon diversity index (trend p = 0.024).

**Fig 3:**
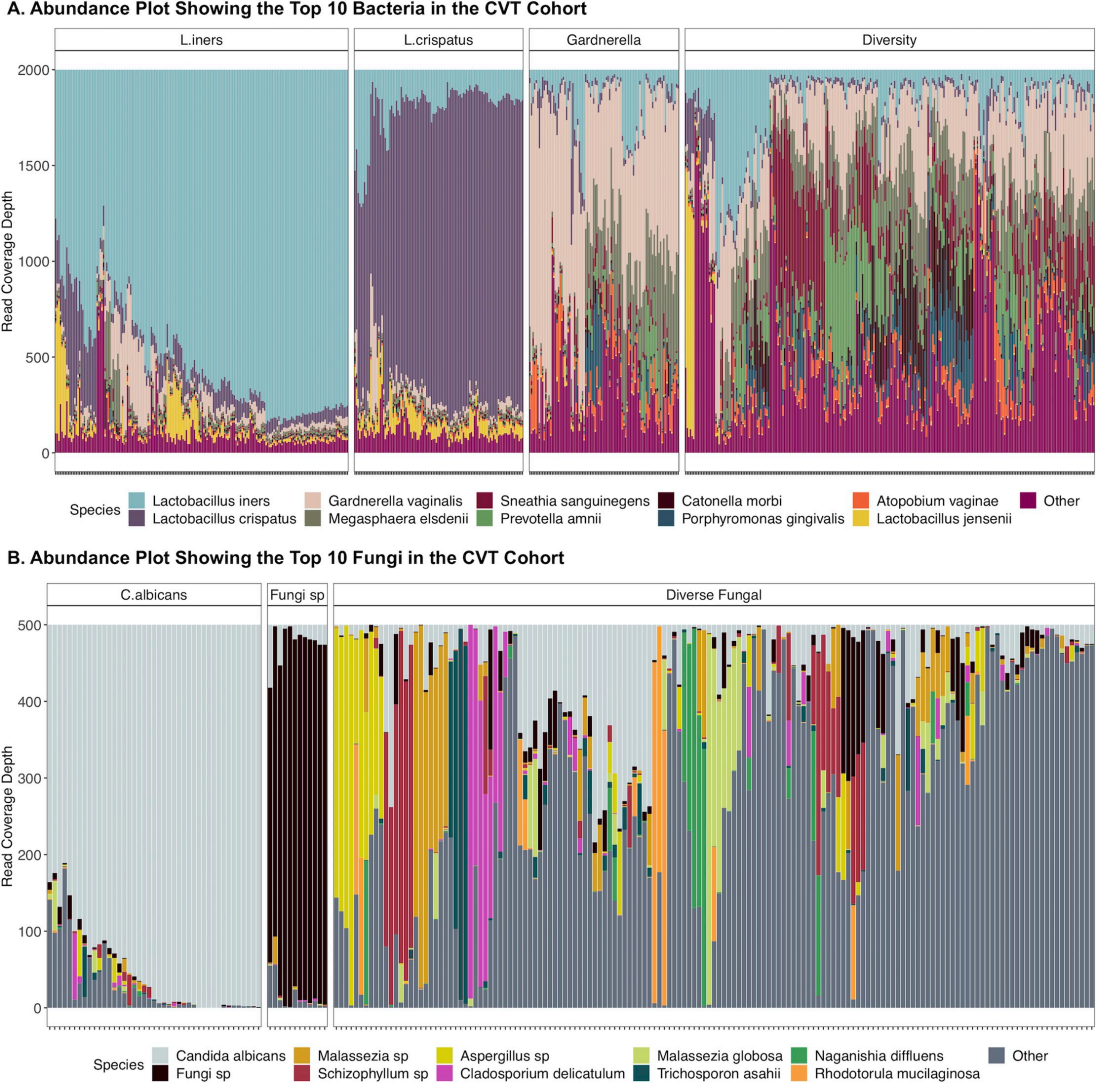
Bacterial and fungal communities within the study cohort. A. The abundance plot represents the bacterial community structure of the study subjects. The operational taxonomic units (OTUs) were collapsed at the species level and the top 10 species are presented. Figure boxes labeled: L. iners, L. crispatus, Gardnerella and Diversity represent the vaginal community state types (CSTs) identified using hierarchical clustering. B. Heatmap showing the top 20 fungal species identified within the study subjects. *C*. *albicans* has the highest mean abundance. There were three vaginal fungal community states identified using hierarchical clustering as indicated by the separate boxes.

To evaluate the overall structure of the cervicovaginal microbiome, we performed hierarchical clustering on all available samples (n = 539) (**Fig 3A**). This analysis revealed four distinct bacterial community state types (CSTs). Two CSTs were dominated by species of the genus *Lactobacillus* (*Lactobacillus iners*, 143/539, 26.5% and *Lactobacillus crispatus*, 83/539, 15.4%), one CST by *Gardnerella vaginalis* (94/539, 17.4%), and the other CST did not contain a major group but had a highly diverse microbiome (219/539, 40.6%).

**Fig 3B** shows results of hierarchical clustering based on the detected fungal species. *Candida albicans* was the dominant fungal taxa. In terms of fungal clusters, there appears to be a single clade dominated by *C*. *albicans* (43/208, 20.7%), one dominated by an unidentified fungal species (12/208, 5.8%) and one that is composed of a diverse fungal community (153/208, 73.6%).

**Fig 4:**
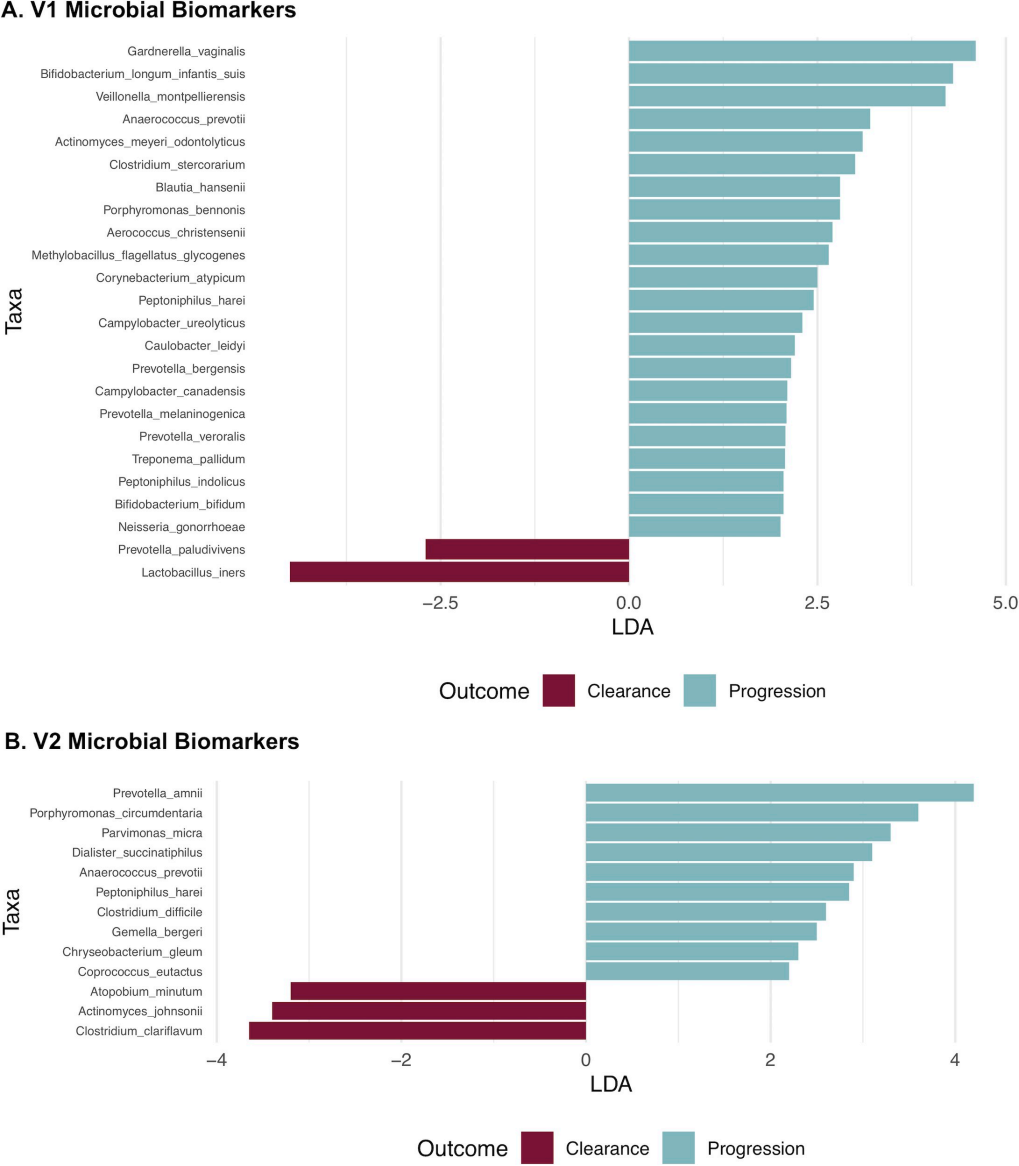
Bacteria associated with progression to CIN2+ identified using LEfSe. HR-HPV bacterial biomarkers for visit 1 (panel A) and visit 2 (panel B), comparing clearance vs. progression, were identified using the LEfSe tool. Only the significant bacterial taxa (LDA>2.0) are shown for both visits.

Analysis of bacterial taxonomic categories of the microbiome associated with persistence/progression vs. clearance revealed a total of 24 taxa that were significant (LDA>2.0) at V1. *G*. *vaginalis* was the bacterial species with the highest positive correlation to progression (**Fig 4A)**, while *L*. *iners* was the most positively associated taxon with clearance based on relative abundance. Amongst V2 samples there were a total of 13 significant taxa identified (three taxa associated with clearance and 10 with progression) (**Fig 4B**). At V2, bacteria that are commonly associated with bacterial vaginosis, such as *Prevotella amnii* and *Anaerococcus prevotii*, were significantly correlated with progression. We used a generalized linear model (GLM) to validate LEfSe biomarkers with adjustments for key covariates (age, smoking status, HPV16 and visit CST) (**S1 Table**).

Fungal biomarker discovery revealed five fungal taxa associated with HPV progression (**S1A Fig**). The average of the five combined fungal taxa were detected at rates of 0.59%, 3.55% and 3.76% for the clearance, persistence and progression outcomes, respectively (p = 0.0080, **S1B Fig**). However in the adjusted GLM analysis, none of the fungal biomarkers were determined to be significant at either visit (**S2 Table**).

To evaluate whether some common function of bacteria might be associated with HR-HPV outcomes, we used a functional analysis of gene groups imputed from the microbiome data as described in the methods. This analysis revealed 8 functional pathways at KEGG Level 2 significantly associated with progression to CIN2+ (**S3 Table**). Of the 8 identified pathways, only 2 had a mean read coverage of >1% and were considered for further analysis. The two identified pathways were “Xenobiotics Biodegradation and Metabolism” pathway, which was positively associated with progression (p = 0.0020) and the Cell Motility pathway, which was negatively associated with progression (p = 0.019). These two pathways were significantly correlated (Pearson correlation = -0.80, **S2 Fig**), and we chose to use Cell Motility in multivariate modeling since it produced a more stable GLM estimate (**S3 Table**).

### Microbiome and HR-HPV natural history: GLM modeling

**Fig 5:**
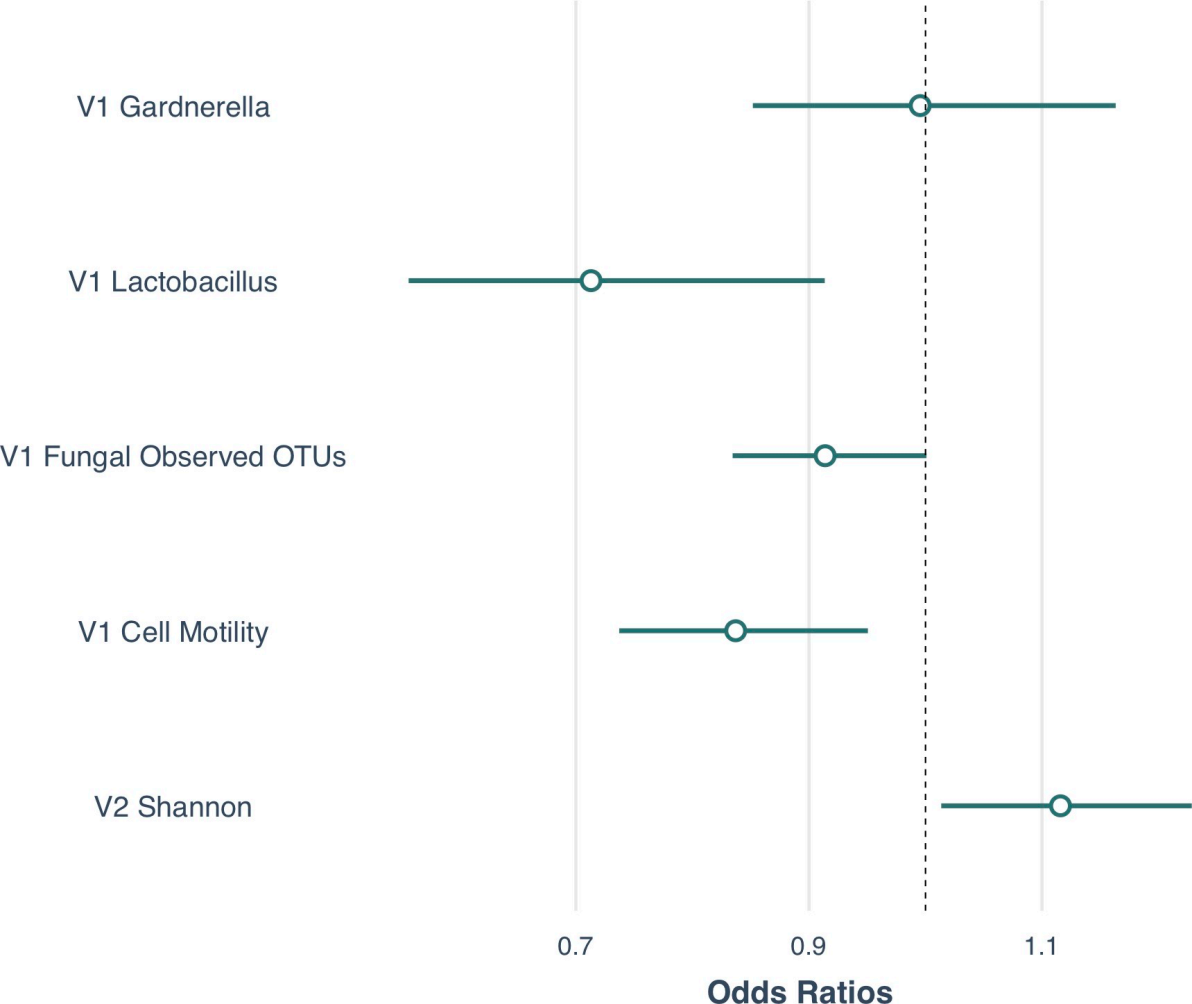
Generalized Linear Model (GLM) results showing the odds ratios of key microbial components in association with progression to CIN2+. The forest plot shows the results of variables evaluated in the univariate analysis that were then entered into a GLM. The model shows ORs (small circle) and 95% confidence interval (line extending on either side of the circle) of the microbial features associated with clearance/progression at either Visit 1 (V1) and/or Visit 2 (V2). The main variables included V1 *Gardnerella*, V1 *Lactobacillus*, V1 Fungal Observed OTUs and V1 Cell Motility and V2 Shannon diversity. The model was adjusted for age, bacterial CSTs, smoking and HPV16 infection status. 95% CIs that did not cross the Odds Ratio of 1.0 (dotted vertical line) are considered statistically significant.

To investigate the contribution of components of the microbiome over time, we used a GLM in order to adjust for known covariates of CIN2+ progression that may influence the relationship of the CVM and progression to CIN2+ (e.g. age, smoking, HPV16 and CST). We utilized a GLM with a binary outcome (clearance/progression) and the significant microbial features identified in preceding sections as predictors. Specifically, we used the abundance of *Gardnerella* at V1, the abundance of *Lactobacillus* at V1, the Observed fungal species diversity at V1, the imputed Cell Motility pathway at V1 and the microbial diversity at V2. The model was adjusted for age, CST, smoking and HPV16 infection status. **Fig 5** shows the model estimates of the resulting GLM analysis. The multivariate analysis revealed a significant protective effect of V1 *Lactobacillus* (genus) abundance, OR = 0.41 (0.22–0.79), V1 fungal species diversity, OR = 0.90 (0.82–1.00) and imputed V1 Cell Motility pathway OR = 0.75 (0.62–0.92). In addition, the model showed that the V2 microbial diversity was a significant risk factor for CIN2+ progression, OR = 1.17 (1.02–1.29).

**Fig 6:**
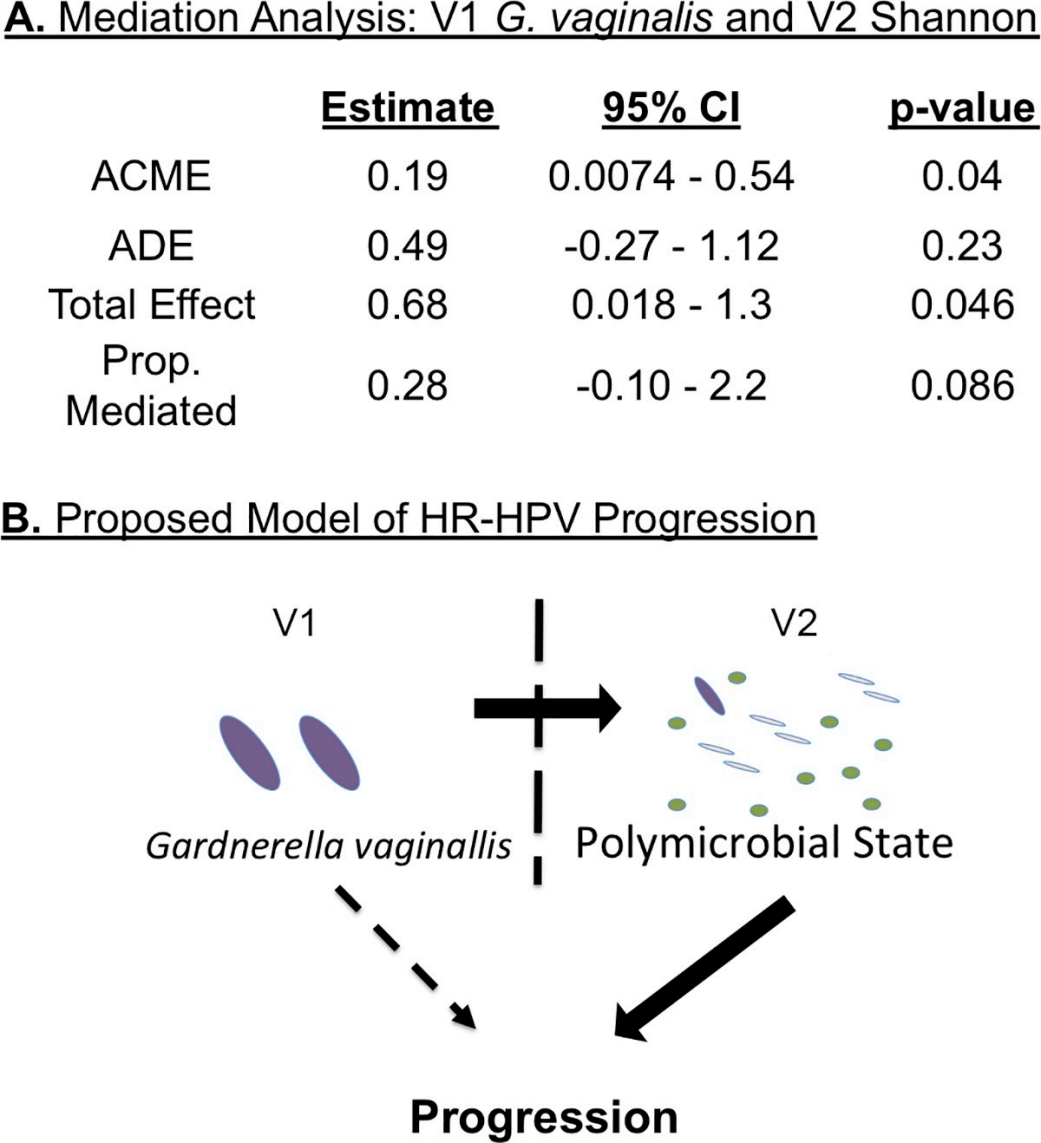
Diversity model for HPV progression with mediation analysis. Panel A shows the results of the mediation analysis that focus on V1 *Gardnerella* and V2 Shannon diversity. Top row shows Average Causal Mediation Effect (ACME) which is the full mediation effect of V2 Shannon diversity after adjusting for the direct effect of V1 *Gardnerella* on case status. The second row shows Average Direct Effect (ADE) which is the direct effect of *Gardnerella* on the clearance/progression outcome after accounting for the mediation effect of V2 Shannon diversity. The third row shows the Total Effect which is the direct, unadjusted effect, of *Gardnerella* on case outcome. The last row shows the Proportion (Prop.) Mediated, which is the proportion of the model that is mediated by V2 Shannon diversity. Based on GLM modeling, we propose the above model (Panel B) in which V1 *Gardnerella* causes an expansion of bacterial diversity at V2, which acts as a risk factor for the progression of a HR_HPV infection into a CIN2+ lesion.

Following the multivariate analysis, we wanted to explore the reason for V1 *Gardnerella* being insignificant despite being the top microbial risk factor in differential abundance in all previous analyses (**Fig 4, S3 Fig** and **S1 Table**). Thus, we performed a mediation analysis to determine if V1 *Gardnerella* was acting by inducing the elevated diversity at V2 (**Fig 6A and 6B**). This analysis showed that after adjustment for V1 *Gardnerella* there was a significant association of V2 Shannon diversity with progression to CIN2+, p = 0.04. The Average Direct Effect (ADE) showed that V1 *Gardnerella* wasn’t significant after adjustment for the V2 Shannon diversity, p = 0.23 supporting our mediation hypothesis (**Fig 6A**).

Power for detection of the effects of each microbiome component was performed using the *lmSupport* package. Specifically each of the microbiome components (i.e., V1 *Gardnerella*, V1 *Lactobacillus*, V1 Fungal Observed OTUs, V1 Cell Motility, and V2 Shannon diversity) was tested separately to assess power with adjustment for age, CST, HPV16 status and smoking. **S4 Table** shows the results of the power calculation. Given large effect sizes for V1 *Lactobacillus*, V1 Fungal Observed OTUs, V1 Cell Motility, and V2 Shannon diversity we calculated that these could be detected with a power >98%, while the V1 *Gardnerella* had a power of 82% at the 0.05 alpha level.

## Discussion

Previous cross-sectional studies analyzing the association between the cervicovaginal microbiome and HPV infection outcomes have consistently identified *Gardnerella* as a key biomarker associated with CIN2+. This has been reported in studies that utilized both next-generation sequencing [39, 41] and other methods of microbiome analyses [52, 43]. We present data that *Gardnerella* is in fact associated with CIN2+ lesions, but rather than directly causing the CIN2+ lesion, appears to induce a higher diversity CVM over time as measured at V2, which in turn mediates the observed effect of *Gardnerella* in HR-HPV disease progression. Although it is not clear how a state of polymicrobialism in the presence of a persistent HR-HPV infection leads to the development of epithelial dysplasia, recent studies on the microbiome’s role in other cancers suggests that the answer lies in the establishment of a microbial microenvironment, perhaps a biofilm. For instance, it has been shown that certain cancers (e.g., colorectal cancer and prostate cancer) have distinct microbial communities at the tumor site that are associated with tumor development [44, 45]. In fact, other data from our lab using cervical biopsy tissue samples indicates that there are distinct microbial differences as cervical cancer progresses to advanced FIGO stages (manuscript in preparation). This idea is further supported by data indicating that cervical precancerous lesions that regress, compared to those that progress to cancer, harbor a different immune microenvironment [46]. The local interplay between the microbiome and the local host immune system may be important to understanding the progression of HR-HPV infection to cervical cancer.

The protective microbial biomarkers identified at V1 also suggest an association of the microbiome and host innate and acquired immunity in progression to CIN2+. Specifically, the protective effect of bacterial Cell Motility may be due to the known phenomena of bacterial flagella activating host immunity [47–49]. This local activation may facilitate the innate immune system’s ability to clear an active HPV infection. Such stimulation may be critical in HPV control since cervical lesions have been shown to be associated with local immunosuppression through the reduction of factors such as IL-17 [50]. Despite the presence of studies to support this conjecture it should be noted that this type of immune activation needs to be confirmed with rigorous experimental precision.

*Gardnerella*, as discussed above, continuously emerges as a risk factor for CIN2+ development and progression. Based on our findings and published data, the association may be tied to the ability of *Gardnerella* to be immunosuppressive in the cervicovaginal region [51]. Whereas, it seems that the presence of commensal bacteria (e.g. *Lactobacillus*) with the ability to stimulate a local immune response may be contributing factors to the clearance of incident HR-HPV infections. Moreover, the presence of bacteria with immunosuppressive attributes, such as *Gardnerella*, may promote viral persistence and progression.

Alternatively, there may be other explanations for the observed associations between the cervicovaginal microbiome and HPV’s natural history. One possible explanation is a host developed or inherited immune deficiency that is a common cofactor for both cervical cancer progression and microbial diversity. For example, elevation of a particular inflammatory cytokine may be both necessary for successful tumor growth and be a causal factor in increasing vaginal microbial diversity. Such a factor may also explain the consistent identification of *Gardnerella*, which is commonly identified as a biomarker for increased diversity in the CVM [52] and a risk factor for CIN2+.

We have identified distinct microbial biomarkers that either protect, or promote the progression of a HR-HPV infection to CIN2+ lesions. In the context of what is known about the cervicovaginal microbiome, it may be that these factors act to suppress (in the case of progression) or activate (in the case of clearance) a localized immune response, which in turn influences the natural history of a HR-HPV infection. However, additional prospective studies are needed to establish a causal link between the cervicovaginal microbiome, the immune system and the natural history of HPV. Nevertheless, our results suggest a marker for identifying women with persistent HR-HPV infection at risk for progression by monitoring the presence of *Gardnerella* and subsequent elevation in microbial diversity. If future studies support a causal role of the cervicovaginal microbiome and disease progression, then it might be possible to manipulate the CVM in a manner to activate a local immune response. It is possible that HPV vaccination might influence the CVM and future research will be needed to evaluate such potential changes.

The strength of this study includes the prospective design and availability of a longitudinal cohort. In addition we used advanced epidemiological methods in a novel way to investigate potential causative factors in cervical intraepithelial neoplasia. Potential weaknesses in this study include the relatively small sample size, homogeneity of the population and the use of only two time points.

In summary, through the use of longitudinal samples from the CVT cohort we investigated and identified key features of the cervicovaginal microbiome potentially associated with progression of HR-HPV infection [28, 53–56] (e.g., *Gardnerella* and subsequent increase vaginal microbial diversity). Additional studies are required to validate the model proposed in this report.

## Materials and methods

### Clinical trial information

The study of cervicovaginal microbiome and HR-HPV natural history was a nested analysis within the previously reported CVT [57] (clinical trials registration NCT00128661). Written informed consent was obtained from all participants in CVT. The trial protocol can be obtained from the original trial publication [57].

### Ethics statement

All CVT participants were adult women between the ages of 18–25 years. All participants were shown a video describing the study design and were then required to provide written consent to continue participating in the trial. Institutional review board approval was obtained for the informed consent forms at both the NCI and in Costa Rica. Registered with Clinicaltrials.gov NCT00128661

### Study population and case definitions

Subjects for this nested study were selected from the placebo arm of a community-based clinical trial of the HPV 16/18 vaccine in Costa Rica that had enrolled women 18 to 25 years of age in 2004–2005 [57]. Women with an incident HR-HPV infection (HPV16, 18, 31, 33, 35, 39, 45, 51, 52, 56, 58, or 59) were selected for analysis. Incident infections were classified based on outcomes from the well-established model of HR-HPV natural history including outcomes of clearance, persistence and progression [58]. Specifically, outcomes related to the incident HR-HPV infection included women who developed a CIN2 or CIN3 (CIN2+) lesion (progression), women with an infection for 2 or more years with the same type in the absence of a CIN2+ diagnosis (persistent), or women who cleared their incident HR-HPV infections within 1 year (clearance). This analysis included 273 women of whom all had available samples at V1 (first visit positive for the studied HPV type) and 266 who had a second sample at V2 (for persistent, visit that was positive for the same type and at least 305 days after V1; progression, closest visit before diagnosis of CIN2+; clearance, following visit that was negative for that type); all had clinical follow-up data. Seven women, one with clearance, and six with persistence either did not have an available V2 sample or the sample failed in lab testing.

### Cervical microbiome characterization

DNA samples [59] were shipped to the Burk Lab on dry ice where the microbiome analysis was performed. DNA had been extracted from cervical brush samples by DDL Diagnostic Laboratory (Voorberg, The Netherlands) where they had been tested for HPV as previously described [60]. Laboratory procedures for the microbiome analyses were performed within a hood (AirClean Systems, Creedmoor, NC) in an isolated room to minimize environmental contamination and water-blank negative controls were used throughout the testing.

Bacterial DNA was amplified using barcoded-primers 16SV4_515F (GTGYCAGCMGCCGCGGTA) and 16SV4_806R (GGACTACHVGGGTWTCTAAT) that amplify the V4 variable region of the 16S rRNA gene [61]. This region has been demonstrated to accurately amplify and resolve vaginal bacteria [62]. PCR reactions were performed with 17.75 μl of nuclease-free PCR-grade water (Lonza, Rockland, ME), 2.5 μl of 10X Buffer w/ MgCl2 (Affymetrix, Santa Clara, CA), 1 μl of MgCl_2_ (25 mM, Affymetrix, Santa Clara, California, USA), 0.5 μl of dNTPs (10 mM, Roche, Basel, Switzerland), 0.25 μl of AmpliTaq Gold DNA Polymerase (5 U/μl, Applied Biosystems, Foster City, California), 0.5 μl of HotStart-IT FideliTaq (2.5 U/μl, Affymetrix, Santa Clara, CA), 1 μl of each primer (5 μM), and 0.5 μl of sample DNA. Thermal cycling conditions consisted of initial denaturation at 95°C for 5 min, followed by 15 cycles at 95°C for 1 min, 55°C for 1 min, and 68°C for 1 min, followed by 15 cycles at 95°C for 1 min, 60°C for 1 min, and 68°C for 1 min, and a final extension for 10 min at 68°C on a GeneAmp PCR System 9700 (Applied Biosystems, Foster City, CA).

The fungal DNA ITS1 region was amplified using barcoded-primers ITS1_48F (ACACACCGCCCGTCGCTACT) and ITS1_217R (TTTCGCTGCGTTCTTCATCG) as previously described [63]. PCR reactions were performed with 8.25 μl of nuclease-free PCR-grade water (Lonza), 2.5 μl of 10X Buffer w/ MgCl_2_ (Affymetrix), 1 μl of MgCl_2_ (25 mM, Affymetrix), 0.5 μl of dNTPs (10 mM, Roche), 0.25 μl of AmpliTaq Gold DNA Polymerase (5 U/μl, Applied Biosystems), 0.5 μl of HotStart-IT FideliTaq (2.5 U/μl, Affymetrix), 1μl of each primer (5 μM), and 10 μl of sample DNA. Thermal cycling conditions consisted of initial denaturation of 95°C for 3 min, followed by 35 cycles at 95°C for 30 sec, 55°C for 30 sec, and 68°C for 2 min, followed by a final extension for 10 min at 68°C on a GeneAmp PCR System 9700 (Applied Biosystems).

For both amplicon experiments, 20 negative controls were randomly mixed amongst samples. Negative controls were created using nuclease-free PCR-grade water (Lonza) as described above instead of extracted DNA.

Barcoded-PCR products were combined for each amplicon type and the DNA fragments (~356 bp for 16S V4 and 400–600 for ITS1) were isolated by gel purification using a QIAquick Gel Extraction Kit (Qiagen, Hilden, Germany). Purified PCR products were quantified using a Qubit 2.0 Fluorometic High Sensitivity dsDNA Assay (Life Technologies, Carlsbad, CA) prior to library construction using a KAPA LTP Library Preparation Kit (Kapa Biosystems, Wilmington, MA). Size integrity of the amplicons was validated with a 2100 Bioanalyzer (Agilent Technologies, Santa Clara, CA). High-throughput amplicon sequencing of 2x300 paired-end reads was conducted on an Illumina MiSeq (Illumina, San Diego, CA).

### Bioinformatics

Illumina reads were trimmed to remove bases that had a PHRED score of <25 using prinseq-lite V0.0.4 [64]. Quality trimmed reads were then demultiplexed using Novobarcode [65]. Paired-end reads were joined using PANDAseq with default settings [66]. The merged reads were processed through the VSEARCH quality-filtering pipeline [67] to dereplicate the sequences, reduce noise and remove chimeric reads.

For bacterial 16S V4 rRNA gene reads, closed-reference OTU picking was performed using VSEARCH [67] with a custom database that contained sequences from the GreenGenes database [68] the Human Oral Microbiome Database (HOMD) [69] and cervicovaginal microbiome 16S reference sequences retrieved from NCBI [70]. Representative sequences were aligned using PyNAST [71] and taxonomy was assigned using VSEARCH [67].

PICRUSt was used to impute microbial functional gene content and to collapse identified genes into functional pathways [72]. Pathways that were associated with HR-HPV clearance were identified through the use of a generalized linear model (GLM) based on statistical significance (p<0.05) and relative abundance (1% or higher across all reads).

For fungal ITS1 reads, open-reference OTU picking was performed using VSEARCH [67] and the UNITE database [73]. Taxonomy of representative fungal sequences was assigned using BLAST [74].

Phyloseq [75] was used to import BIOM data for 16S and ITS assays into R separately, followed by the determination of Shannon and Chao1 alpha diversity. For all analyses, bacterial data was subsampled for 2,500 reads. For fungal analyses subsampling was performed at 500 reads. Biomarker discovery analysis was performed using the LEfSe tool [76]. Linear discriminant analysis (LDA) scores greater than 2.0 are considered to be significant [76]. Microbial community state types (CSTs) were assigned on the basis of hierarchical clustering of the 20 most abundant OTUs. Prior to clustering, OTUs were agglomerated at the species level or the lowest identified taxonomic level. Clustering was performed using the wardD2 algorithm using Euclidian distances.

### Statistical analysis

R v3.4.2 [77] was used for statistical analyses. The Kruskal-Wallis test was used to assess significance of continuous data. Linear regression was used to assess the significance of variables associated with the ordered outcome states of a HR-HPV infection (1). Logistic regression was performed using the GLM function and a binomial family generalized linear model in R. For categorical data, dummy variables were created and each individual factor level was tested in a univariate GLM analysis. Models were adjusted for age, smoking, HPV16 and CSTs. Smoking status was determined through a questionnaire and incorporated into the model as ordered categories: never smoked, former smoker and current smoker [40]. Power of GLM results was computed using the *lmSupport* package [78].

We performed a statistical mediation analysis to test whether V1 *Gardnerella* (an independent variable) could be acting by inducing a subsequent elevated microbiome diversity at V2 (mediator variable) that influences the outcome of HR-HPV progression using the package *mediation* [79]. The outcome model we used was binary clearance/progression. Models were adjusted for age, CST, smoking status and HPV16 infection status. In the results we present the mediation effect (average causal mediation effects (ACME)), which is the total effect of V2 Shannon diversity and V1 *Gardnerella* minus the direct effect of V1 *Gardnerella*. Additionally, we estimate the direct of effect (presented using the average direct effect (ADE)) of V1 *Gardnerella* on the binary outcome clearance/progression, minus the effect of the V2 Shannon diversity mediator; the total effect, which is the sum between the indirect effect of the V2 Shannon diversity and the direct effect of the V1 *Gardnerella*; and the proportion mediated which is the ratio of the ACME and total effect estimates.

### Investigators in the International Agency for Research on Cancer/World Health Organization

Where authors are identified as personnel of the International Agency for Research on Cancer/ World Health Organization, the authors alone are responsible for the views expressed in this article and they do not necessarily represent the decisions, policy or views of the International Agency for Research on Cancer / World Health Organization.

### Investigators in the Costa Rica HPV Vaccine Trial (CVT) group

Proyecto Epidemiológico Guanacaste, Fundación INCIENSA, San José, Costa Rica—Bernal Cortés (specimen and repository manager), Paula González (LTFU: co-principal investigator), Rolando Herrero (CVT: co-principal investigator), Silvia E. Jiménez (trial coordinator), Carolina Porras (co-investigator), Ana Cecilia Rodríguez (co-investigator).

United States National Cancer Institute, Bethesda, MD, USA—Allan Hildesheim (co-principal investigator & NCI co-project officer), Aimée R. Kreimer (LTFU: co-principal investigator & NCI co-project officer), Douglas R. Lowy (HPV virologist), Mark Schiffman (CVT: medical monitor & NCI co-project officer), John T. Schiller (HPV virologist), Mark Sherman (CVT: QC pathologist), Sholom Wacholder (statistician).

Leidos Biomedical Research, Inc., Frederick National Laboratory for Cancer Research, Frederick, MD, USA (HPV Immunology Laboratory)—Ligia A. Pinto, Troy J. Kemp

Georgetown University, Washington, DC, USA—Mary K. Sidawy (CVT: histopathologist)

DDL Diagnostic Laboratory, Netherlands (HPV DNA Testing)—Wim Quint, Leen-Jan van Doorn, Linda Struijk.

University of California, San Francisco, CA, USA—Joel M. Palefsky (expert on anal HPV infection and disease diagnosis and management), Teresa M. Darragh (pathologist and clinical management)

University of Virginia, Charlottesville, VA, USA—Mark H. Stoler (QC pathologist)

## Supporting information

S1 Table Bacterial GLM confirmation. (CSV)

S2 Table Fungal GLM confirmation. (CSV)

S3 Table PICRUSt functional pathways. (CSV)

S4 Table Power calculation. (XLSX)

S1 Fig Fungal taxa associated with HPV natural history. Panel A shows specific fungal taxa, identified as being significant with the three HR-HPV outcomes. Values that are higher than LDA score of 2.0 are considered to be significant. Panel B shows the main fungal taxa identified in panel A with their relative abundances. The box represents the median value (with the 25–75% confidence interval as the box and the 95% confidence interval with the whiskers) for the taxa in each outcome (shown in the three separate panels and labeled at the top of the panel). There is a statistically significant increase in the sum of the five progression associated taxa when going from clearance to persistence to progression, p = 0.0080. The y-axis is the log of the relative abundance.
(PDF)

S2 Fig Correlation between cell motility and xenobiotic metabolism. The two pathways identified after PICRUSt that were significantly different between clearance and progression were analyzed to determine their correlation to each other. Plot shows a significant negative correlation (Pearson correlation = -0.80) between the two pathways and thus they are highly correlated.
(PDF)

S3 Fig 16S bacterial Shannon diversity index and *Gardnerella* across categorical cytology groups. Panel A shows the 16S bacterial Shannon diversity index in each cytology group. Panel B shows the relative abundance of *Gardnerella* across the cytology groups. Significance is shown above bar plots as indicated in the figure at the top right.
(PDF)
