# Supplementary material for: Cervicovaginal microbiome and natural history of HPV in a longitudinal study
Source: PLoS Pathog. 2020 Mar 26;16(3):e1008376. doi: 10.1371/journal.ppat.1008376 (PMC7098574; doi:10.1371/journal.ppat.1008376)

A. Fungal Taxa Associated with HR-HPV Outcomes

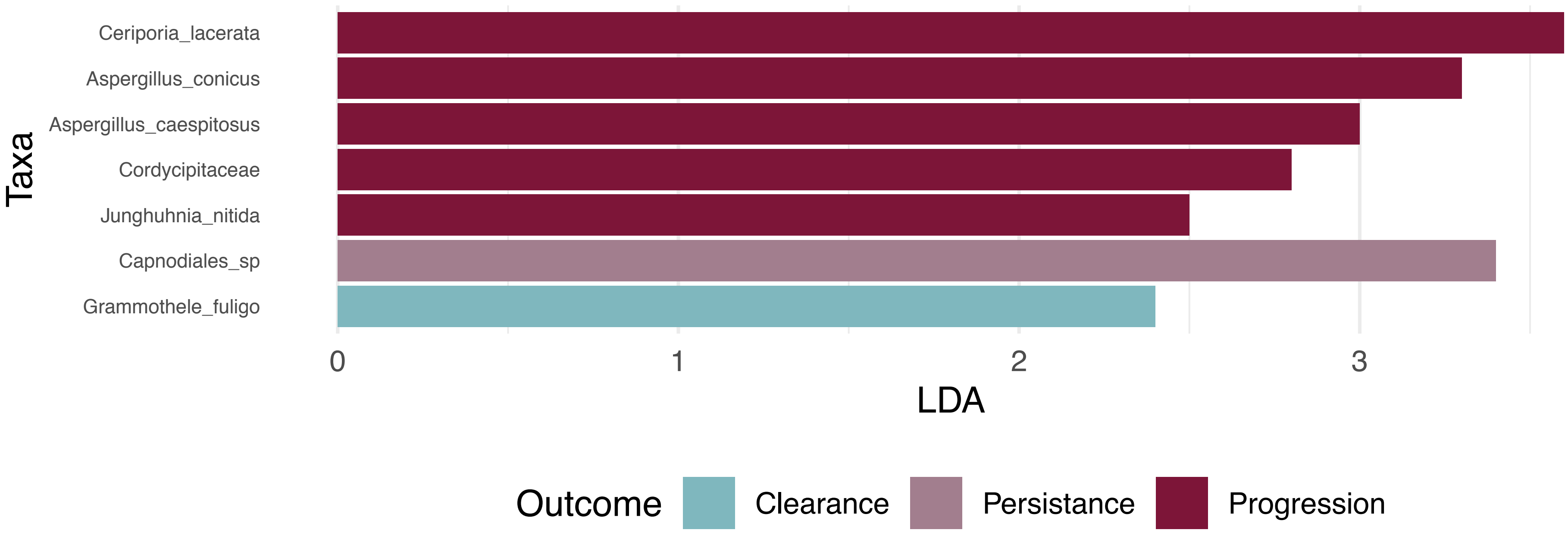

B. Relative Abundance of Progression associated Taxa Across HR-HPV Outcomes

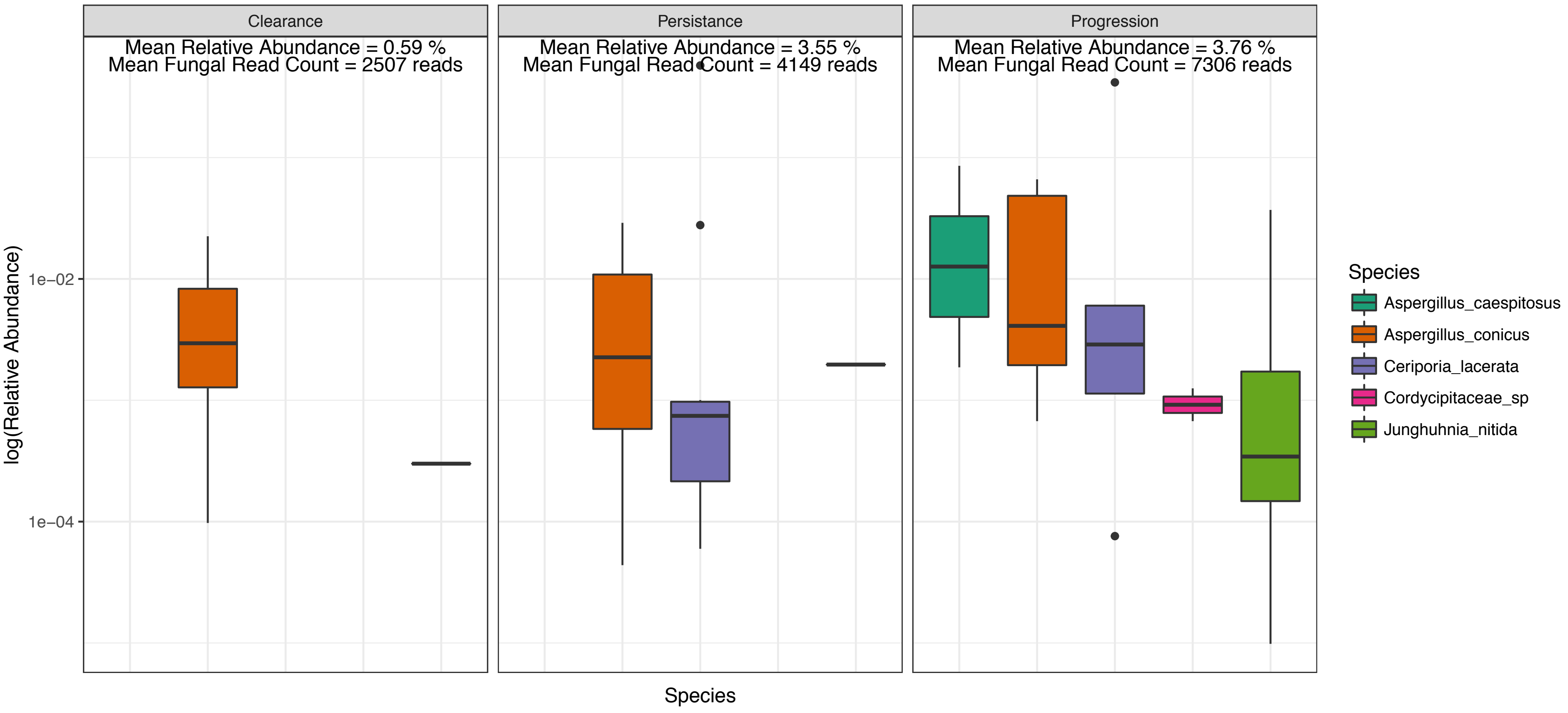

Supplement: S1 Fig — Panel A shows specific fungal taxa, identified as being significant with the three HR-HPV outcomes. Values that are higher than LDA score of 2.0 are considered to be significant. Panel B shows the main fungal taxa identified in panel A with their relative abundances. The box represents the median value (with the 25–75% confidence interval as the box and the 95% confidence interval with the whiskers) for the taxa in each outcome (shown in the three separate panels and labeled at the top of the panel). There is a statistically significant increase in the sum of the five progression associated taxa when going from clearance to persistence to progression, p = 0.0080. The y-axis is the log of the relative abundance. (PDF) [file ppat.1008376.s005.pdf]
