# Supplementary figures and images for: Cervicovaginal microbiome and natural history of HPV in a longitudinal study
Source: PLoS Pathog. 2020 Mar 26;16(3):e1008376. doi: 10.1371/journal.ppat.1008376 (PMC7098574; doi:10.1371/journal.ppat.1008376)

# Significant Pathway Predictor Correlation

Pearson correlation =  $-0.8$

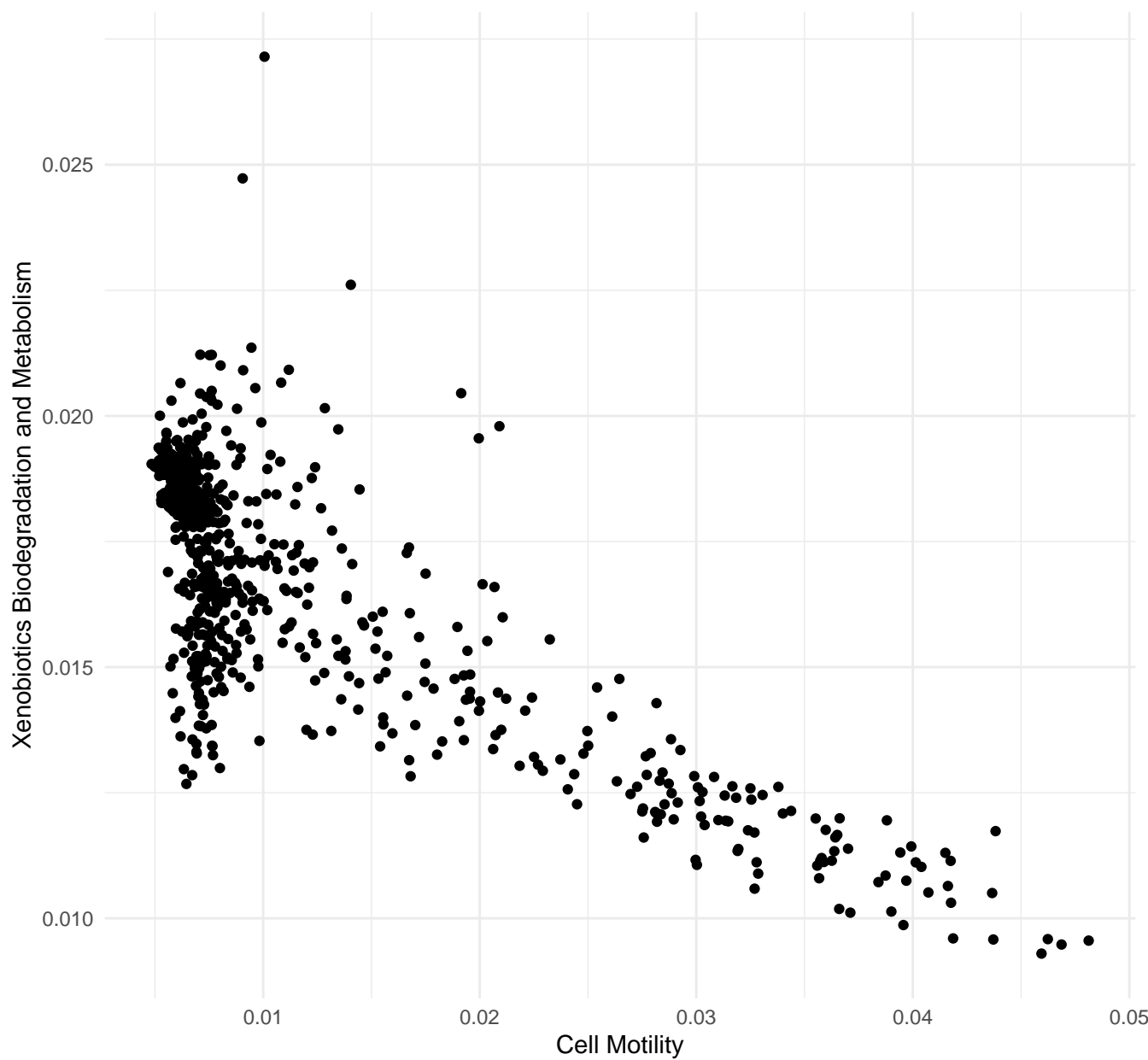

Supplement: S2 Fig — The two pathways identified after PICRUSt that were significantly different between clearance and progression were analyzed to determine their correlation to each other. Plot shows a significant negative correlation (Pearson correlation = -0.80) between the two pathways and thus they are highly correlated. (PDF) [file ppat.1008376.s006.pdf]

A.

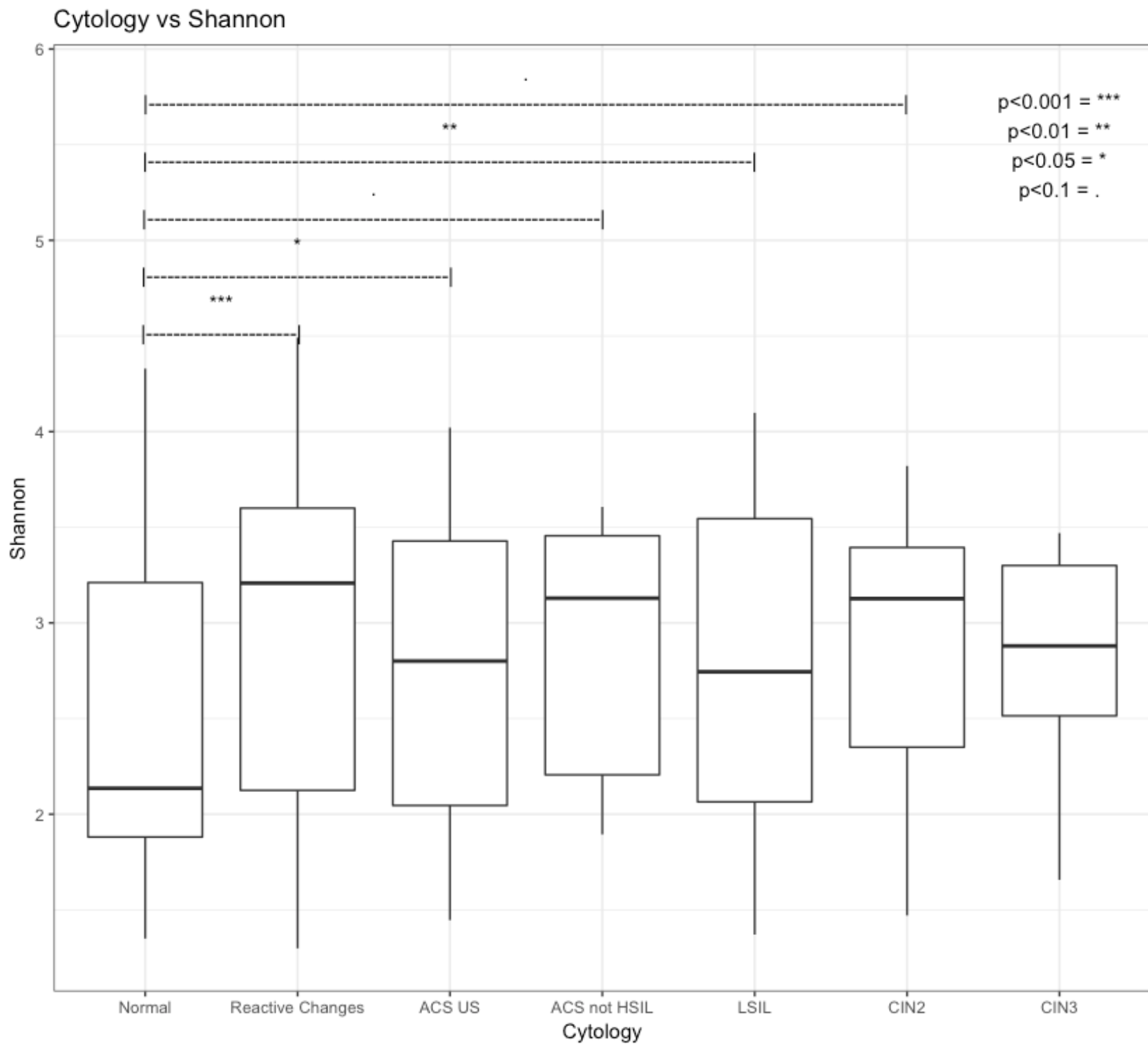

B.

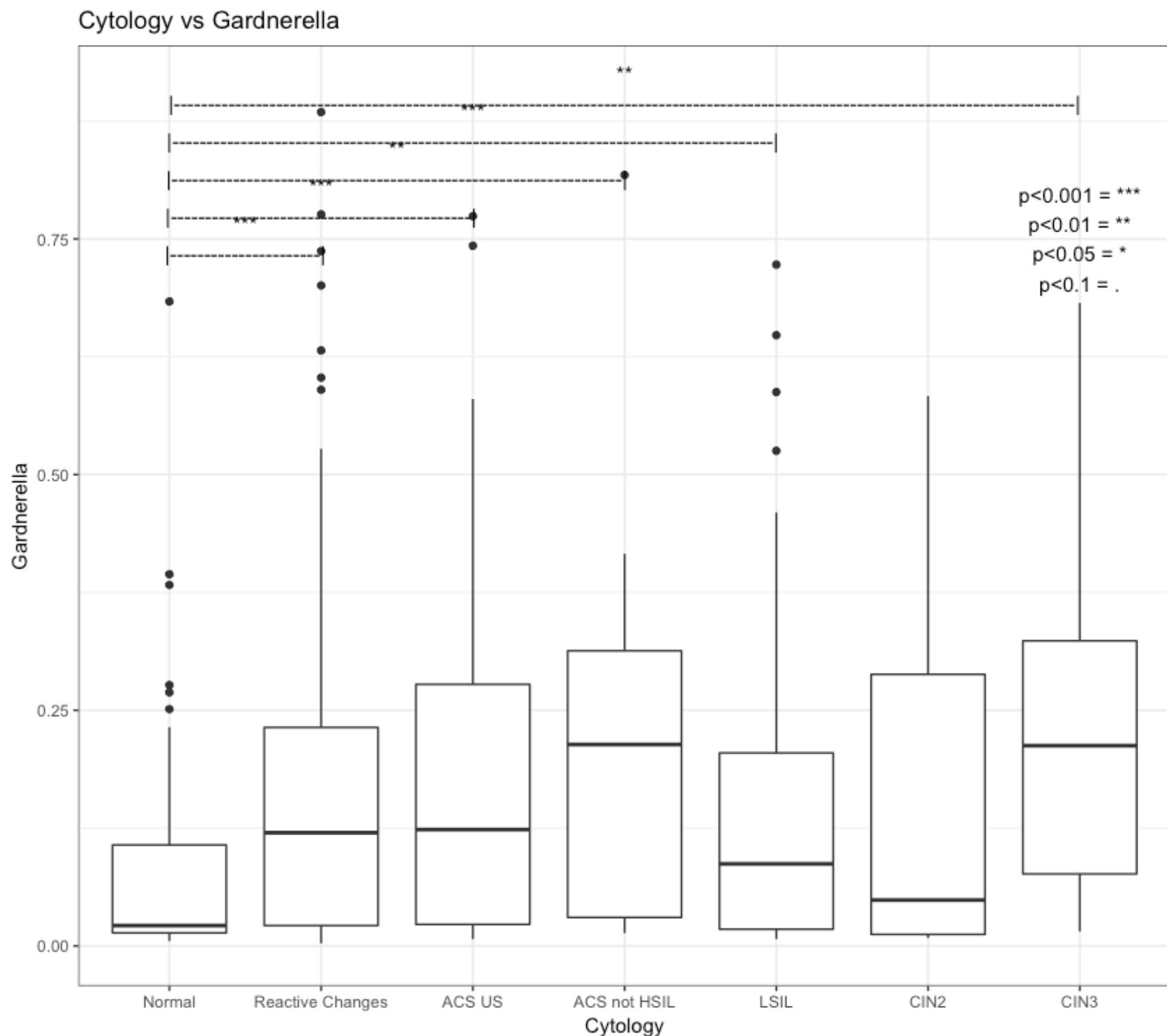

Supplement: S3 Fig — Panel A shows the 16S bacterial Shannon diversity index in each cytology group. Panel B shows the relative abundance of Gardnerella across the cytology groups. Significance is shown above bar plots as indicated in the figure at the top right. (PDF) [file ppat.1008376.s007.pdf]
